# A 36-month follow-up of decline in activities of daily living in individuals receiving domiciliary care

**DOI:** 10.1186/s12877-015-0047-7

**Published:** 2015-04-14

**Authors:** Anne-Sofie Helvik, Lisbeth D Høgseth, Sverre Bergh, Jūratė Šaltytė-Benth, Øyvind Kirkevold, Geir Selbæk

**Affiliations:** Department of Public Health and General Practice, Faculty of Medicine, Norwegian University of Science and Technology (NTNU), Postbox 8905, NO7491 Trondheim, Norway; Norwegian National Advisory Unit on Ageing and Health, Vestfold Hospital Trust, Tønsberg, Norway; St Olavs University Hospital, Trondheim, Norway; Centre for Old Age Psychiatric Research, Innlandet Hospital Trust, Ottestad, Norway; Institute of Clinical Medicine, Campus Ahus, University of Oslo, Oslo, Norway; HØKH, Research Centre, Akershus University Hospital, Lørenskog, Norway; Faculty of Health, Care and Nursing, Gjøvik University College, Gjøvik, Norway; Akershus University Hospital, Lørenskog, Norway

**Keywords:** Older people, MCI, Dementia, CDR, Neuropsychiatric symptoms, Cognitive functioning

## Abstract

**Background:**

There have been few studies of how personal and instrumental activities of daily living (P-ADL and I-ADL) develop over time in older people receiving domiciliary care. This study aimed at assessing variables associated with the development of P-ADL and I-ADL functioning over a 36-month follow-up period, with a particular focus on cognitive functioning.

**Method:**

In all, 1001 older people (≥70 years) receiving domiciliary care were included in a longitudinal study with three assessments of P-ADL and I-ADL functioning during 36 months. P-ADL and I-ADL were assessed using the Lawton and Brody’s Physical Self-Maintenance Scale and Instrumental Activities of Daily Living Scale, respectively. Mini Mental State Examination (MMSE), diagnosis of dementia and MCI, neuropsychiatric symptoms and use of psychotropic medication were also evaluated during the three assessments. Baseline demographic and general medical health information and information of being a nursing home resident at follow-up were recorded. Linear mixed models were estimated.

**Results:**

There was a significant decline in P-ADL and I-ADL functioning throughout the follow-up. A lower MMSE sum-score, diagnosed MCI and dementia, a higher level of neuropsychiatric symptoms and the use of antipsychotics and antidepressants recorded at each assessment were associated with a decline in both P-ADL and I-ADL functioning. Furthermore, a decline in P-ADL and I-ADL functioning at follow-ups was associated with being male, a higher baseline age and in poorer medical health as well as residing in a nursing home at follow-up.

**Conclusion:**

P-ADL and I-ADL functioning in older people worsened over time. The worsening was associated with lower MMSE sum-score, diagnosed MCI and dementia, poorer medical health, neuropsychiatric symptoms, use of psychotropic medication and being transferred to nursing home care. Clinicians should pay close attention to the assessment and treatment of these factors to help older people maintain their level of functioning for as long as possible.

## Background

In older people, cognitive state and activities of daily living (ADL) functioning are important for mortality, institutionalization, and quality of life [1-3]. ADL describes practical everyday tasks that are necessary for sustaining basic and more complex self-care [2,4,5].

The association between cognitive decline and decline in ADL functioning in older people is well-known from numerous of studies covering a wide range of countries [6-11] that used both cross-sectional [10-13] and longitudinal design [6-9,14-19]. The cross-sectional studies generally found that participants with cognitive impairment were more likely to have ADL impairment, while the longitudinal studies generally found that poorer cognitive functioning at baseline was associated with reduced ADL functioning during follow-up. However, with one exception [18], these longitudinal studies of older people living in a community included only two assessments. Moreover, the sample populations in the longitudinal community studies differed widely: one included only women [17], some were independent of health status [7,14], one only included healthy people [16], two only included those without limitations in ADL functioning [9,15], and three only included those with Alzheimer’s disease at the baseline [8,18,20]. However, to our knowledge, there have been no longitudinal studies of older community-dwelling people who are dependent on formal care, such as in-home nursing care or domiciliary care.

Cognitive functioning is one of several factors important in the decline in ADL functioning [6]. Other major risk factors are physical limitations, such as general medical health, weight loss, falls, chronic conditions, musculoskeletal impairment, higher number of prescribed drugs, and hearing loss and/or vision deficits [6,21]. Moreover, the decline in ADL functioning interacts with psychological factors and/or environmental constraints [6,22], and is associated with advanced age [6]. Furthermore, some studies have reported gender differences in how ADL functioning declines [3,6]. Even though it has been widely studied, the multifactorial and complex etiology for the decline in ADL functioning [6] is not yet fully understood. To better understand the dynamics of the mechanisms for the decline in ADL functioning, a prospective study would benefit from including more than two assessments of community-dwelling individuals. A distinction between personal activities of daily life (P-ADL) and instrumental activities of daily life (I-ADL) make it easier to understand the mechanisms of ADL decline. P-ADL includes personal hygiene, bathing, dressing, eating, and moving inside the house. I-ADL, on the other hand, includes making meals, cleaning, shopping, household activities, managing finances, administering medication, and using transportation. Furthermore, to improve the understanding of the relation between cognition and ADL decline, adjustments for conditions such as dementia, the neuropsychiatric symptom load, and use of psychotropic medication would be helpful. Community-dwelling older people receiving domiciliary care are a vulnerable group that is likely to need future nursing home placement. Following such a vulnerable group of older people may contribute to a new understanding of the mechanisms of the decline in ADL functioning over time.

We conducted a study to examine the association between cognitive functioning, both at the baseline and at two follow-ups over a 36-month period, and to examine the development in P-ADL and I-ADL functioning assessed by the Physical Self-Maintenance Scale (P-ADL sum-score) and the Instrumental Activities of Daily Living Scale (I-ADL sum-score) [4], respectively. Our goal was to see if adjusting for a number of other variables known to have an influence on the P-ADL and I-ADL functioning in older people would modify these associations. We hypothesized that the P-ADL and I-ADL functioning in older community-dwelling people receiving domiciliary care at the baseline will decline over time, and that this decline is associated with a worsening of cognitive functioning, prevalence of dementia, higher neuropsychiatric symptom load, use of psychotropic medication, and type of formal care received at each follow-up assessment.

## Methods

### Design

This was a 36-month prospective study with three assessments. The baseline assessment took place between August 2008 and December 2010. The two follow-up assessments took place after 18 and 36 months.

### Participants

A representative sample of older people (≥70 years) receiving domiciliary care was recruited from 19 municipalities in five counties in the eastern part of Norway. Both rural and urban municipalities of various sizes were invited to participate in the study. A random selection of older recipients of domiciliary care with a next of kin who saw them at least once a week was made, regardless of the amount and kind of service received. Of 1,796 eligible people, 795 declined to participate [23]. Those who declined were more often women than men (73.0% vs. 68.1%, p = 0.004) and were older (mean age 85.0 years with standard deviation (SD) 6.2 years vs. mean age 83.4 (SD = 5.7) years, p < 0.001) than those who were included in the study [23]. A total of 1,001 older people receiving domiciliary care were included.

### Measures

The levels of personal and instrumental functioning (the dependent variables) were reported by the next of kin at all assessments and classified using Lawton and Brody’s Physical Self-Maintenance Scale (P-ADL) and Instrumental Activities of Daily Living Scale (I-ADL) [4]. The P-ADL sum-score is based on six items (range 6–30) with higher scores indicating a lower level of functioning, while the I-ADL sum-score is based on eight items (range 0–8) with a higher score indicating better I-ADL functioning [4,24]. Lawton & Brody’s Physical Self-Maintenance Scale and the Instrumental Activities of Daily Living Scale are among the shorter recommended P-ADL and I-ADL scales [25], which have been frequently used in Norwegian and Scandinavian studies [26,27] and are suitable for ADL assessments in community-dwelling older people, as well as in nursing home residents [28,29].

Cognitive functioning was evaluated at each assessment by the Mini-Mental State Examination (MMSE) [30], Informant Questionnaire on Cognitive Decline in the Elderly (IQ-CODE) [31], and the Clock Drawing Test (CDT) [32]. The MMSE is a 30-point interviewer-administered screening test for cognitive impairment, where a higher score indicates better cognitive functioning [30]. The IQ-CODE is an interview with the closest proxy, who assesses observed changes in cognitive functioning over the past ten years. “No change” is scored 3 on a scale ranging 0–5. Values < 3 indicate improvement and values > 3 indicate deterioration [31]. The CDT is rated to a score of 5 for a “perfect” clock; errors from minor to severe are given a score from 4 to 1. Inability to make any reasonable representation of a clock is given a zero score [32]. These measures have been translated and adapted to Norwegian conditions [33,34].

The severity of the dementia was evaluated at each assessment by the Clinical Dementia Rating Scale (CDR; [35]). The CDR assesses the severity of dementia in six domains (memory, orientation, judgment and problem solving, community affairs, home and hobbies, and personal care). A total score of 0 (no dementia), 0.5 (possible), 1 (mild), 2 (moderate), and 3 (severe dementia) is calculated by means of an algorithm that weight priority to memory [35,36]. CDR has been translated, validated [37], and used in several settings in Norway [38-41].

Two physicians (SB & GS) with extensive experience in research and clinical old age psychiatry independently diagnosed dementia according to the ICD-10 criteria and mild cognitive impairment (MCI) according to the Winblad criteria [42] using all the available information at each assessment. In cases of disagreement, a third clinical expert was consulted and a consensus was reached. Neuropsychiatric symptoms were evaluated at all assessments using the Neuropsychiatric Inventory (10-item NPI) [43] in a translated and validated Norwegian version [44]. The 10-item version covers the following symptoms: delusion, hallucination, euphoria, agitation/aggression, disinhibition, irritability/lability, depression/dysphoria, anxiety, apathy/indifference, and aberrant motor behavior. The next of kin rated each symptom based on its occurrence the previous four weeks. We identified three sub-syndromes of the NPI based on a principal component analysis with direct oblimin rotation. The components were extracted based on the Kaiser criterion (factors with eigenvalues under 1 are dropped) and inspection of the screen plot. We termed the sub-syndromes “Agitation,” “Psychosis,” and “Affective symptoms.” “Agitation” encompassed characteristics of agitation/aggression, euphoria, disinhibition, aberrant motor behavior, and irritability; “psychosis” was composed of the characteristic of delusions and hallucinations. “Affective symptoms” covered the characteristics of depression, anxiety, and apathy. The characteristic of agitation/aggression loaded on the “Psychosis” sub-syndrome as well, but in line with previous research and clinical experience, we chose to include it in the “Agitation” sub-syndrome.

Psychotropic medications were grouped according to the ATC code, as antipsychotics (N05A except lithium), antidepressants (N06A), anxiolytics (N05B), hypnotics/sedatives (N05C), and anti-dementia medication (N06D) (yes versus no). The information was collected from the medical record of each individual at the initial assessment.

The General Medical Health Rating Scale was used at the baseline to evaluate comorbidity. This is a four-point global scale for scoring the degree of somatic illness from very good (1) to very poor (4). The scale considers each patient’s number of general medical conditions, the severity of these conditions, and the use of medication due to the conditions [45].

Demographic information including age, gender, marital status at inclusion, and municipality of residence, was collected as a part of the general baseline examination.

Formal level of care over time was recorded as living at home with domiciliary care, or living in a nursing home.

### Procedure

A research nurse coordinated the project and cooperated with the health workers, who were assessors in each of the municipalities. The assessors, mostly nurses, social educators, and occupational therapists, interviewed participants and their next of kin in the 19 municipalities. Before the baseline data collection, all assessors went through a two-day course with training in how to use the assessment scales. Interviews with each participant and their next of kin were performed simultaneously in their own homes by two separate assessors. A one-day training program was conducted prior to each follow-up assessment.

Both written and oral study information was given to the participants and their next of kin. Written informed consent was obtained from both the participant and their next of kin before the interviews were conducted. In those lacking the capacity to give consent, the closest family proxy gave informed consent on behalf of their next of kin. The project was approved by the Regional Committee for Medical and Health Research Ethics for Eastern Norway (S-08111b), the Norwegian Social Science Data Services (NSD) (07–2008SI), and the Directorate for Health and Social Affairs (08/2984).

### Data analysis

Continuous socio-demographic and clinical characteristics were presented as means and standard deviations (SD), while frequencies and proportions were used for categorical characteristics. An intra-class correlation coefficient (ICC) was calculated for both outcomes, P-ADL and I-ADL, to quantify a proportion of intra-municipality variation. There was a significant cluster effect on the municipality level found in both variables, confirming a hierarchical structure in the data. Moreover, the data were collected at three times, comprising repeated measurements for each patient. Therefore, a linear mixed model with random intercepts for patients and nursing homes was estimated (SAS MIXED procedure). Fixed effects for both linear and second-order time components were included. The second-order time component was only left in the model if it was significant. Trend estimates were further adjusted for the (main) independent variable, the degree of cognitive functioning (MMSE sum-score). An interaction term between the degree of cognitive functioning (MMSE) and time was included into the model. For each outcome (P-ADL and I-ADL sum-score), two different models with respect to adjustments were estimated. In both models, adjustment variables included demographic characteristics (age, gender, and marital status), dementia, general somatic health, neuropsychiatric symptoms, and use of psychotropic medications. The first model contained adjustment variables that were assessed at the baseline. The second model included demographic characteristics and general somatic health, also assessed at the baseline, while dementia, neuropsychiatric symptoms, and use of psychotropic medications were entered as longitudinal variables. Changes in level of care from domiciliary care to nursing home care were also included in the second model. The results of the regression analysis were tabulated as coefficients with the corresponding 95% confidence intervals (CI) and p-values. The P-ADL and I-ADL sum-scores at each assessment, which were estimated as the average value of the MMSE sum-score from the baseline assessment, were illustrated graphically.

Analyses were performed in SAS v9.3 and SPSS v22. P-values below 0.05 were considered statistically significant. All tests were two-sided.

## Results

### Sample characteristics

**Figure 1:**
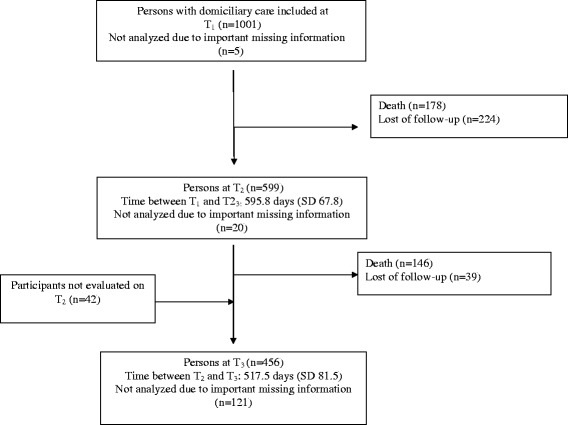
Flow chart of participants from baseline (T_1)_ to last follow-up (T_3)_, with a mean (SD) follow-up time at each assessment.

At the baseline, the mean (SD) age was 83.4 (5.7) years (see Table 1). In all, 683 (68.2%) of the participants were women and 703 (70.2%) were single or were a widow/widower. The mean (SD) baseline MMSE sum-score was 24.5 (4.8), and at the first and second follow-up, the mean (SD) sum-score of MMSE was 23.1 (6.2) and 22.4 (7.4), respectively. Of the 1,001 participants at the baseline (T1), 599 (59.8%) and 456 (45.5%) participants were available for the second and third assessments (i.e., T2 and T3), respectively (see Figure 1). At follow-up, 86 participants (14.4%) and 114 participants (25.2%) had been admitted to a nursing home by the second and third assessments, respectively.

**Table 1:** **Characteristics of study sample at baseline (N = 1001)**

		**Total**	
*Demographic*			
Women	N *(%)*	683	*(68.2)*
Age (year)	Mean *(SD)*	*83.4*	*(5.7)*
Single as marital status	N *(%)*	703	*(70.2)*
*General somatic health* ^1^			
Good	N *(%)*	155	*(15.6)*
Fair	N *(%)*	392	*(39.2)*
Poor	N *(%)*	346	*(34.6)*
Very poor	N *(%)*	106	*(10.6)*
*Cognitive functioning*			
MMSE	Mean *(SD)*	24.5	*(4.8)*
*Diagnoses*			
MCI	N *(%)*	277	*(27.7)*
Dementia	N *(%)*	415	*(41.5)*
*Neuropsychiatric sub-syndrome score*			
Agitation	Mean *(SD)*	1.7	*(4.6)*
Psychosis	Mean *(SD)*	0.5	*(2.0)*
Affective	Mean *(SD)*	2.9	*(5.3)*
*Use of psychotropic medication*			
Antipsychotics	N *(%)*	34	*(3.4)*
Antidepressants	N *(%)*	154	*(15.4)*
Anxiolytics	N *(%)*	86	*(8.6)*
Sedatives	N *(%)*	218	*(21.8)*
Antidementia	N *(%)*	56	*(5.6)*

*MMSE =* Mini-Mental State Examination, *MCI* = Mild cognitive impairment.

^1^Does not sum up to 1001 due to missing information.

### Change of P-ADL functioning over the follow-up period

The mean P-ADL sum-score increased over time (see Table 2), indicating a worsening in P-ADL functioning, confirmed by a linear mixed model demonstrating a significant trend in time.

**Table 2:** **P-ADL and I-ADL score at three time points**

**Time point**	**Min, Max**	**Mean (SD)**	**ICC municipality (%)**
P-ADL			
T1	6, 23	9.2 (3.5)	
T2	6, 25	10.3 (4.4)	5.6
T3	6, 30	11.4 (5.2)	
I-ADL			
T1	0, 8	5.5 (2.3)	
T2	0, 8	4.8 (2.5)	8.0
T3	0, 8	4.6 (2.7)	

Higher P-ADL score indicates poorer functioning.

Lower I-ADL score indicates poorer functioning.

*T1* = Baseline, *T2* = The second assessment, *T3* = The third assessment.

### The association between cognitive functioning and P-ADL functioning

The association between cognitive functioning, assessed with the MMSE sum-score, and P-ADL functioning over time was studied in two multivariate models. In the first adjusted model (see Table 3), there was still a significant linear time trend in P-ADL functioning. Furthermore, poorer cognitive functioning at the baseline was associated with a stronger decline in P-ADL functioning over time.

**Table 3:** **Model 1: Effects of cognitive impairment (MMSE) measured at baseline on P-ADL level over time estimated by linear mixed model with random effects for intercepts and time** ^**1**^

	**Unadjusted regression coefficients**		**Adjusted regression coefficients**	
	**Coeff. (95% CI)**	**p-value** ^**2**^	**Coeff. (95% CI)**	**p-value** ^**2**^
Effect of main variables^3^				
Time	0.27 (0.21; 0.33)	**<0.001**	0.29 (0.23; 0.34)	**<0.001**
MMSE at T1	−0.28 (−0.33; −0.23)	**<0.001**	−0.12 (−0.18; −0.05)	**<0.001**
MMSE *Time	−0.008 (−0.01; −0.006)	**<0.001**	−0.009 (−0.01; −0.006)	**<0.001**
Effect of additional variables at T1^4^				
*Socio –demographic information*				
Women	−0.91 (−1.30; −0.52)	**<0.001**	−0.57 (−0.92; −0.21)	**0.002**
Age (years)	0.11 (0.07; 0.14)	**<0.001**	0.06 (0.04; 0.10)	**<0.001**
Single	−0.83 (−1.23; −0.43)	**<0.001**	−0.31 (−0.68; 0.06)	0.101
*General somatic health*				
Very poor	4.32 (3.60; 5.05)	**<0.001**	3.32 (2.66; 3.97)	**<0.001**
Poor	2.88 (2.35; 3.41)	**<0.001**	2.04 (1.56; 2.52)	**<0.001**
Fair	1.59 (1.09; 2.10)	**<0.001**	0.97 (0.52; 1.42)	**<0.001**
Good	Reference		Reference	
*Diagnoses*				
Dementia	3.98 (3.58; 4.38)	**<0.001**	1.17 (0.53; 1.80)	**<0.001**
MCI	0.98 (0.55; 1.40)	**<0.001**	−0.10 (−0.53; 0.34)	0.667
No	Reference		Reference	
*Neuropsychiatric sub-syndrome score*				
Agitation	0.15 (0.11; 0.19)	**<0.001**	0.04 (−0.008; 0.09)	0.106
Psychosis	0.44 (0.35; 0.53)	**<0.001**	0.18 (0.08; 0.28)	**<0.001**
Affective	0.15 (0.12; 0.19)	**<0.001**	0.03 (−0.01; 0.06)	0.162
*Use of psychotropic medication*				
Antipsychotics	1.55 (0.60; 2.50)	**0.001**	0.55 (−0.27; 1.37)	0.187
Antidepressants	1.12 (0.63; 1.61)	**<0.001**	0.51 (0.06; 0.95)	**0.026**
Anxiolytics	1.00 (0.34; 1.65)	**0.003**	0.20 (−0.38; 0.77)	0.506
Sedatives	0.49 (0.03; 0.94)	**0.036**	0.17 (−0.23; 0.56)	0.410
Cognitive enhancers	1.91 (1.14; 2.67)	**<0.001**	−0.61 (−1.31; 0.09)	0.088

*MMSE =* Mini-Mental State Examination, *MCI* = Mild cognitive impairment, *T1* = baseline.

^1^All analyses were adjusted for the cluster effect due to municipality belonging.

^2^Bold text indicate p-value<0.05.

^3^The coefficients (95%CI) of the main independent variables (time, MMSE, and interaction between time and MMSE) unadjusted and adjusted for other independent variables.

^4^The coefficients (95%CI) for single independent variables from the model containing time component.

Table 4 presents the results from the second model, which explores the association between the degree of cognitive functioning and the P-ADL functioning assessed simultaneously. As presented in the first adjusted model (Table 3), there was a decline in P-ADL functioning throughout the follow-up period. Furthermore, a greater decline in P-ADL functioning over time was found in those with a lower MMSE. In addition, being male, older, and in poorer general somatic health at the baseline were all associated with worse P-ADL functioning throughout the observation period. Among the independent variables evaluated at all three assessments, dementia (vs. no cognitive impairment), a higher agitation sub-syndrome score and affective sub-syndrome score, as well as use of antipsychotic medication and not using anti-dementia medication were associated with lower P-ADL functioning. Being a nursing home resident at the time of the second and third assessments was associated with significantly poorer P-ADL functioning than those living at home during the same assessments.

**Table 4:** **Model 2: Effects of cognitive impairment (MMSE) measured at three time points on P-ADL level at the same time points estimated by linear mixed model with random effects for intercepts and time** ^**1**^

	**Unadjusted regression coefficients**		**Adjusted regression coefficients**	
	**Coeff. (95% CI)**	**p-value** ^**2**^	**Coeff. (95% CI)**	**p-value** ^**2**^
Effect of main variables^3^				
Time	0.13 (0.08; 0.17)	**<0.001**	0.08 (0.04; 0.13)	**<0.001**
MMSE (at 3 time points)	−0.30 (−0.34; −0.26)	**<0.001**	−0.13 (−0.19; −0.08)	**<0.001**
MMSE*Time	−0.004 (−0.006; −0.002)	**<0.001**	−0.002 (−0.004; −0.0005)	**0.012**
Effect of additional variables at T1^4^				
*Socio –demographic information*				
Women	0.91 (−1.30; −0.52)	**<0.001**	−0.49 (−0.82; −0.16)	**0.003**
Age (years)	0.11 (0.07; 0.14)	**<0.001**	0.04 (0.01; 0.07)	**0.003**
Single	−0.83 (−1.23; −0.43)	**<0.001**	−0.17 (−0.51; 0.16)	0.311
*General somatic health*				
Very poor	4.32 (3.60; 5.05)	**<0.001**	2.99 (2.39; 3.59)	**<0.001**
Poor	2.88 (2.35; 3.41)	**<0.001**	1.77 (1.34; 2.21)	**<0.001**
Fair	1.59 (1.09; 2.10)	**<0.001**	0.65 (0.24; 1.06)	**0.002**
Good	Reference		Reference	
Effect of additional variables at 3 time-points^4^				
*Diagnoses*				
Dementia	4.42 (4.04; 4.80)	**<0.001**	1.31 (0.76; 1.86)	**<0.001**
MCI	0.96 (0.54; 1.39)	**<0.001**	0.18 (−0.22; 0.58)	0.369
No	Reference		Reference	
*Neuropsychiatric sub-syndrome score*				
Agitation	0.23 (0.19; 0.27)	**<0.001**	0.07 (0.03; 0.10)	**<0.001**
Psychosis	0.41 (0.33; 0.48)	**<0.001**	0.06 (−0.01; 0.13)	0.098
Affective	0.17 (0.13; 0.20)	**<0.001**	0.04 (0.01; 0.08)	**0.007**
*Use of psychotropic medication*				
Antipsychotics	2.92 (2.07; 3.78)	**<0.001**	0.81 (0.11; 1.50)	**0.023**
Antidepressants	1.61 (1.15; 2.07)	**<0.001**	0.33 (−0.06; 0.72)	0.095
Anxiolytics	1.30 (0.71; 1.90)	**<0.001**	0.20 (−0.28; 0.68)	0.410
Sedatives	0.81 (0.37; 1.24)	**<0.001**	0.15 (−0.19; 0.50)	0.388
Cognitive enhancers	1.78 (1.08; 2.49)	**<0.001**	−1.09 (−1.68; −0.49)	**<0.001**
Effect of additional variable at T2 & T3^4^				
Nursing home care	6.45 (5.87; 7.03)	**<0.001**	2.84 (2.19; 3.49)	**<0.001**

*MMSE=* Mini-Mental State Examination, *MCI*= Mild cognitive impairment, *T1*= baseline, *T2* and *T3* = follow-ups.

^1^All analyses were adjusted for the cluster effect due to municipality belonging.

^2^Bold text indicate p-value<0.05.

^3^The coefficients (95%CI) of the main independent variables (time, MMSE, and interaction between time and MMSE) unadjusted and adjusted for other independent variables.

^4^The single coefficients (95%CI) of the main independent variables from the modeling containing time components.

**Figure 2:**
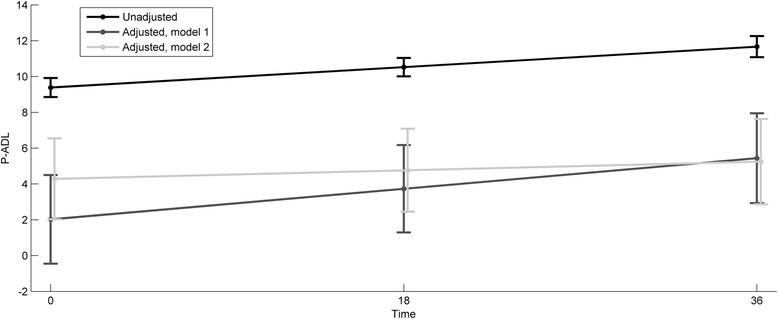
Development of P-ADL sum-score in time, unadjusted, adjusted in Model 1 and adjusted in Model 2.

Figure 2 illustrates the unadjusted P-ADL development over the follow-up period, as well as the P-ADL development adjusted in two different ways (the two P-ADL models). There was an upward trend in the P-ADL values over time that was independent of the adjustments made, i.e., the P-ADL functioning declines. The degree of decline in P-ADL functioning during the follow-up period was moderated in the multivariate models.

### Change of I-ADL functioning over the follow-up period

The mean I-ADL sum-score at the three assessments (see Table 2) indicated worsening in I-ADL functioning, which was confirmed by the second-order growth model. The second-order time trend showed that the I-ADL functioning declined (lower score) over time at the beginning of the follow-up period and flattened after the second assessment.

### The association between cognitive functioning and I-ADL functioning

The association between cognitive functioning, assessed with the MMSE sum-score, and I-ADL functioning over time was studied in two adjusted models. The association between time and cognitive functioning at the baseline and I-ADL functioning over time was assessed in the first adjusted model of I-ADL functioning (Table 5). There was a significant second-order time trend in I-ADL functioning. In addition, poorer cognitive functioning at the baseline was associated with poorer I-ADL functioning over time. The association between degree of cognitive functioning at baseline and I-ADL functioning became weaker over time, implying that I-ADL functioning declined over time, but not at a constant speed; the decline was faster in the beginning and slowed down later.

**Table 5:** **Model 1: Effects of cognitive impairment (MMSE) measured at baseline on I-ADL level over time estimated by linear mixed model with random effects for intercepts and time** ^**1**^

	**Unadjusted regression coefficients**		**Adjusted regression coefficients**	
	**Coeff. (95% CI)**	**p-value** ^**2**^	**Coeff. (95% CI)**	**p-value** ^**2**^
Effect of main variables^3^				
Time	−0.13 (−0.17; −0.09)	**<0.001**	−0.13 (−0.17; −0.10)	**<0.001**
Time* Time	0.0007 (0.00007; 0.001)	**0.029**	0.0006 (0.00001; 0.001)	**0.046**
MMSE at T1	0.24 (0.21; 0.26)	**<0.001**	0.07 (0.04; 0.10)	**<0.001**
MMSE *Time	0.003 (0.002; 0.004)	**<0.001**	0.003 (0.002; 0.004)	**<0.001**
Effect of additional variables at T1^4^				
*Socio –demographic information*				
Women	0.82 (0.59; 1.04)	**<0.001**	0.62 (0.43; 0.80)	**<0.001**
Age (years)	−0.08 (−0.10; −0.06)	**<0.001**	−0.06 (−0.07; −0.04)	**<0.001**
Single	0.71 (0.48; 0.95)	**<0.001**	0.35 (0.16; 0.54)	**<0.001**
*General somatic health*				
Very poor	−2.33 (−2.75; −1.91)	**<0.001**	−1.51 (−1.85; −1.17)	**<0.001**
Poor	−1.72 (−2.03; −1.42)	**<0.001**	−1.05 (−1.29; −0.80)	**<0.001**
Fair	−1.03 (−1.32; −0.74)	**<0.001**	−0.55 (−0.78; −0.32)	**<0.001**
Good	Reference		Reference	
*Diagnoses*				
Dementia	−3.05 (−3.26; −2.84)	**<0.001**	−1.40 (−1.73; −1.08)	**<0.001**
MCI	−0.84 (−1.06; −0.62)	**<0.001**	−0.22 (−0.45; −0.003)	**0.047**
No	Reference		Reference	
*Neuropsychiatric sub-syndrome score*				
Agitation	−0.10 (−0.12; −0.07)	**<0.001**	0.02 (−0.006; 0.04)	0.155
Psychosis	−0.35 (−0.40; −0.30)	**<0.001**	−0.16 (−0.20; −0.11)	**<0.001**
Affective	−0.13 (−0.15; −0.11)	**<0.001**	−0.04 (−0.06; −0.02)	**<0.001**
*Use of psychotropic medication*				
Antipsychotics	−1.29 (−1.83; −0.75)	**<0.001**	−0.49 (−0.91; −0.08)	**0.019**
Antidepressants	−0.77 (−1.06; −0.49)	**<0.001**	−0.34 (−0.57; −0.11)	**0.003**
Anxiolytics	−0.76 (−1.15; −0.38)	**<0.001**	−0.20 (−0.50; 0.10)	0.183
Sedatives	−0.09 (−0.35; 0.17)	0.509	0.07 (−0.13; 0.27)	0.490
Cognitive enhancers	−2.46 (−2.91; −2.02)	**<0.001**	−0.62 (−0.99; −0.25)	**0.001**

*MMSE=* Mini-Mental State Examination, *MCI*= Mild cognitive impairment, *T1*= baseline.

^1^All analyses were adjusted for the cluster effect due to municipality belonging.

^2^Bold text indicate p-value<0.05.

^3^The coefficients (95%CI) of the main independent variables (time, MMSE, and interaction between time and MMSE) unadjusted and adjusted for other independent variables.

^4^The coefficients (95%CI) for single independent variables from the model containing time components.

Table 6 presents the results from the second model of I-ADL functioning, which explored the time trend and the association between cognitive functioning and I-ADL functioning assessed simultaneously. As in the first model (Table 5), there was a significant second-order time trend in I-ADL functioning, i.e., a decline in I-ADL functioning was found throughout the follow-up period, but the I-ADL decline flattened over time. Furthermore, a more severe decline in I-ADL functioning over time was found in those with a poorer MMSE sum-score, and as in the first model, the association between degree of cognitive functioning and I-ADL functioning flattened over time.

**Table 6:** **Model 2: Effects of cognitive impairment (MMSE) measured at three time points on I-ADL level at the same time points estimated by linear mixed model with random effects for intercepts and time** ^**1**^

	**Unadjusted regression coefficients**		**Adjusted regression coefficients**	
	**Coeff. (95% CI)**	**p-value** ^**2**^	**Coeff. (95% CI)**	**p-value** ^**2**^
Effect of main variables^3^				
Time	−0.06 (−0.09; −0.02)	**<0.001**	−0.07 (−0.10; −0.03)	**<0.001**
Time*Time	0.0007 (0.00009; 0.001)	**0.023**	0.0006 (0.0001; 0.001)	**0.016**
MMSE (at 3 time points)	0.25 (0.22; 0.27)	**<0.001**	0.07 (0.04; 0.10)	**<0.001**
MMSE*Time	0.0009 (−0.0001; 0.002)	0.081	0.001 (0.0002; 0.002)	**0.021**
Effect of additional variables at T1^4^				
*Socio –demographic information*				
Women	0.82 (0.59; 1.04)	**<0.001**	0.63 (0.46; 0.80)	**<0.001**
Age (years)	−0.08 (−0.10; −0.06)	**<0.001**	−0.04 (−0.06; −0.03)	**<0.001**
Single	0.71 (0.48; 0.95)	**<0.001**	0.29 (0.11; 0.46)	**0.001**
*General somatic health*				
Very poor	−2.33 (−2.75; −1.91)	**<0.001**	−1.34 (−1.65; −1.03)	**<0.001**
Poor	−1.72 (−2.03; −1.42)	**<0.001**	−0.91 (−1.14; −0.69)	**<0.001**
Fair	−1.03 (−1.32; −0.74)	**<0.001**	−0.43 (−0.64; −0.21)	**<0.001**
Good	Reference		Reference	
Effect of additional variables at 3 time-points^4^				
*Diagnoses*				
Dementia	−3.28 (−3.47; −3.08)	**<0.001**	−1.50 (−1.79; −1.21)	**<0.001**
MCI	−0.76 (−0.98; −0.55)	**<0.001**	−0.26 (−0.47; −0.06)	**0.012**
No	Reference		Reference	
*Neuropsychiatric sub-syndrome score*				
Agitation	−0.13 (−0.15; −0.11)	**<0.001**	0.005 (−0.01; 0.02)	0.634
Psychosis	−0.28 (−0.33; −0.24)	**<0.001**	−0.07 (−0.11; −0.03)	**<0.001**
Affective	−0.14 (−0.15; −0.12)	**<0.001**	−0.05 (−0.07; −0.04)	**<0.001**
*Use of psychotropic medication*				
Antipsychotics	−1.83 (−2.34; −1.33)	**<0.001**	−0.75 (−1.12; −0.39)	**<0.001**
Antidepressants	−0.97 (−1.24; −0.70)	**<0.001**	−0.23 (−0.43; −0.02)	**0.028**
Anxiolytics	−0.95 (−1.30; −0.59)	**<0.001**	−0.29 (−0.54; −0.04)	**0.026**
Sedatives	−0.29 (−0.55; −0.04)	**<0.0251**	0.03 (−0.15; 0.21)	0.768
Cognitive enhancers	−2.38 (−2.79; −1.98)	**<0.001**	−0.46 (−0.78; −0.14)	**0.005**
Effect of additional variable at T2 & T3 ^2^				
Nursing home care	−3.70 (−4.09; −3.31)	**<0.001**	−1.16 (−1.54; −0.79)	**<0.001**

*MMSE=* Mini-Mental State Examination, *MCI*= Mild cognitive impairment, *T1*= baseline, *T2* and *T3* = follow-ups.

^1^All analyses were adjusted for the cluster effect due to municipality belonging.

^2^Bold text indicate p-value<0.05.

^3^The coefficients (95%CI) of the main independent variables (time, MMSE, and interaction between time and MMSE) unadjusted and adjusted for other independent variables.

^4^The single coefficients (95%CI) of the main independent variables from the modeling containing time components.

In addition, being male, older, married and in poorer general medical health at the baseline were associated with worse I-ADL functioning throughout the observation period. Among the independent variables with evaluations at all three assessments in the analysis, MCI and dementia (versus no cognitive impairment), a higher psychosis sub-syndrome score and affective sub-syndrome score, as well as using antipsychotic, antidepressants, anxiolytics, and anti-dementia medication were associated with lower I-ADL functioning. Being a nursing home resident at the time of the second and third assessments was associated with significantly poorer I-ADL functioning at the same assessments compared to those living at home at the same assessments.

**Figure 3:**
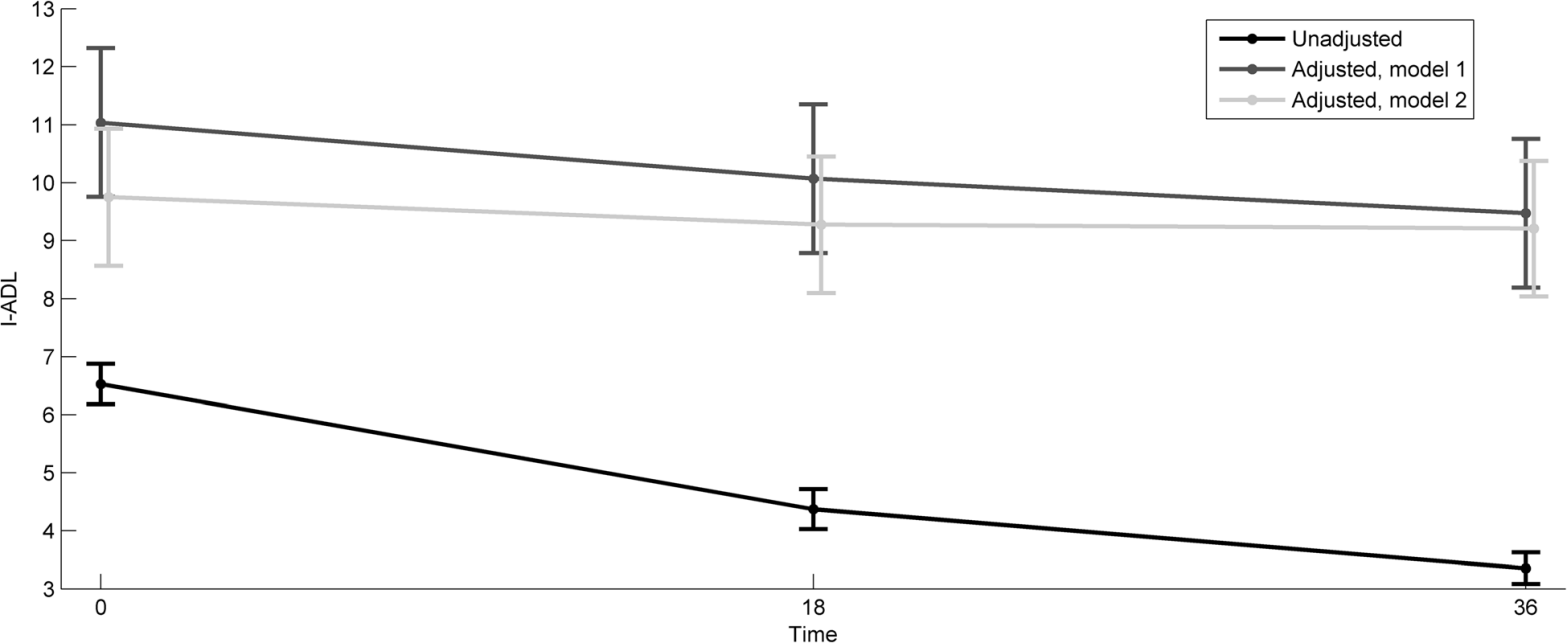
Development of I-ADL sum-score in time, unadjusted, adjusted in Model 1 and adjusted in Model 2.

Figure 3 illustrates the unadjusted I-ADL development over the follow-up period, as well as I-ADL development adjusted in two different ways (the two I-ADL models). Independently of the adjustments made, there is a downward trend in the I-ADL values over time, i.e., the I-ADL functioning declines, but the rate of decline flattened during follow-up. The degree of decline in I-ADL functioning during follow-up was moderated in the multivariate models.

## Discussion

This follow-up study of 1,001 older community-dwelling people (≥70 years) receiving domiciliary care found that reduced cognitive functioning at the baseline and the course of cognitive functioning assessed with MMSE during follow-up were associated with significantly lower P-ADL and I-ADL functioning. Independently of the adjustments made, there was a decline in P-ADL and I-ADL functioning over time. In addition, the rate of decline in I-ADL functioning, but not P-ADL functioning, flattened during the time of follow-up.

Numerous other authors have pointed to a relationship between reduced cognitive functioning and worsening P-ADL in older community-dwelling people [2,6-9,14-19], as well as among older hospitalized patients [46], and in nursing home residents [47]. However, to our knowledge, few have studied the P-ADL development by time and degree of cognitive functioning in older community-dwelling people, including three or more assessments. A recent Swedish community study of older people diagnosed with MCI or Alzheimer’s disease conducted P-ADL assessments three times during a follow-up period of eight years, and their results supported our findings that a decline in P-ADL functioning was explained by both time and degree of cognitive impairment [18]. Our results, as well as the results of the Swedish study, indicate the importance of “tailoring” care for people with cognitive impairment as the cognitive impairment progresses and P-ADL decreases. As previously suggested, tailoring care according to the patients’ increasing needs will likely increase the quality of life for the people involved [48].

In the present study, we assessed P-ADL and I-ADL functioning separately and at all assessments. We found a decline in I-ADL functioning by time and cognitive functioning, as we did for P-ADL functioning, but we also found that the slope of decline in I-ADL functioning flattened at the third assessment. The I-ADL assessment covers more complex activities of daily living than the P-ADL assessment [49]. Impairment in I-ADL functioning may be an earlier sign of cognitive impairment [50], and the decline may flatten at an earlier point of time than the decline in P-ADL functioning. Thus, the shape of decline in P-ADL and I-ADL functioning throughout the assessment period may differ. However, we cannot rule out the possibility that the slope in I-ADL development may be explained by a ceiling effect related to the assessment tool used. However, the results indicate the importance of “tailoring” care for people with cognitive impairment as cognitive impairment progresses and ADL functioning decreases.

Independent of the assessed ADL functioning outcome (I-ADL or P-ADL functioning), we found that lower age and better general somatic health at the baseline were associated with better functioning throughout the assessment period. These results are in accordance with findings from other community studies assessing risk factors for the decline of ADL functioning over time [6]. In the present study, we have used the GMHR Scale to assess somatic health. The scale considers each patient’s number of general medical conditions, the severity of these conditions, and the use of medication due to the conditions. It rates the condition of somatic health from very good (1) to very poor (4) [45]. An equally sound approach to assessing somatic health difficulties could have been the use of more detailed information about somatic comorbidity or a comorbidity index such as the Charlson comorbidity index [51]. It is crucial to adjust for somatic comorbidity regardless of the assessment tool chosen when examining the association between cognitive functioning and ADL decline, since somatic health difficulties may have negative consequences for cognitive functioning over time [28].

In population-based studies, men often reported higher P-ADL and I-ADL functioning than women [3]. In the present study, men had worse P-ADL and I-ADL functioning than women at each assessment. This might be due to the inclusion criteria used in the study, i.e., being a recipient of domiciliary care. Nevertheless, we cannot rule out the possibility that this trend was due to next of kin answering the ADL questions. However, a prospective community study of people with dementia found that women had better I-ADL functioning at the time of follow-up after adjustment for cognitive functioning [20]. Furthermore, a cross-sectional community study found that women reported better I-ADL functioning than men in analyses adjusted for cognitive impairment, [10]. The authors in the latter study explained the finding as a result of traditional culture-related gender roles, i.e., that men were more likely to get help with complex activities than women [10].

In the adjusted analyses, we found that participants with dementia had worse P-ADL and I-ADL functioning at each time point compared to participants without dementia. Furthermore, we found that those with MCI had worse I-ADL functioning, but not P-ADL functioning at the same assessments as those without dementia. This was expected since previous research has found MCI to affect complex ADL (I-ADL) functioning, but not basic self-care (P-ADL) functioning [50], while dementia often affects both complex and basic ADL functioning [52,53].

Interestingly, we found that worsening ADL functioning at each assessment was associated with higher levels of neuropsychiatric symptoms at the same assessment. Higher agitation and affective sub-syndromes scores were associated with worsening P-ADL functioning, while higher psychosis and affective sub-syndromes scores were associated with worsening I-ADL functioning. To our knowledge, no previous studies have explored neuropsychiatric symptoms in relation to ADL functioning in community-dwelling older people. However, a recent study of decline in P-ADL functioning with four assessments in nursing home residents are in line with the present study in reporting that higher neuropsychiatric symptoms were associated with worsening P-ADL functioning [54]. Moreover, the present study found an association between better P-ADL functioning and the use of anti-dementia medication at the same assessment, which is in line with the results of the previously mentioned study of nursing home residents and several randomized trials that found anti-dementia medication important for P-ADL [25,54-56]. Lower I-ADL functioning at each assessment was also associated with the use of anti-dementia medication, antipsychotics, and/or antidepressants, and anxiolytics were associated with lower P-ADL and I-ADL functioning at the same assessment. In contrast to our results, a study of psychogeriatric outpatients with dementia reported that a low dose use of antipsychotics for half a year did not change P-ADL or I-ADL functioning [57]. Unfortunately, we do not have information about the dosages and length of use of the psychotropic drugs in our study. However, as the use of antipsychotic drugs is associated with a range of other serious adverse events, these drugs should be used with great caution and regular evaluation of effects and side effects.

Community-dwelling older people receiving domiciliary care represent a vulnerable group, and at assessments two and three, 86 (14.4%) and 114 (25.2%) participants had become nursing home residents, respectively. Being a receiver of nursing home care at assessment two and three was associated with worsening ADL functioning at the same assessments compared to those receiving care at home. Other studies have also reported that a decline in ADL is associated with nursing home admission [58,59]. In addition, nursing home admission is not known to improve or stabilize ADL functioning, but rather lowers ADL functioning during the first period after admission [60].

While the study has a number of advantages, such as using well-known, internationally accepted P-ADL and I-ADL assessment scales, a high number of baseline participants, use of a direct measure of cognitive functioning, and adjustments for a high number of variables of potential importance to the outcome, such as medical health, demographic variables, and diagnosed MCI and dementia, it nevertheless has some limitations that need to be addressed.

First, the associations found in our study should be interpreted with caution since our design does not allow for inferences about causality.

Secondly, participants differed in mean age and gender distribution from the original population, many of whom refused participation at the baseline [23]. Even though the participants were randomly selected, the great number of those who declined to participate may not be random and to some extent may have biased the representativeness of the study population. Thus, caution should be exercised in generalizing the study results.

Thirdly, assessments of P-ADL and I-ADL functioning in older people in a longitudinal study with three follow-ups over a three years period are statistically challenging. Repeated measurements of the same individuals imply dependency in data. Having a large number of drop-outs due to death and other reasons, leads to a varying number of observations per individual and generates data imbalances. Furthermore, a number of the independent variables in the adjusted models vary with time. However, the linear mixed model handles any degree of imbalance in data by including all available data [61]. It also accounts for the correlations among repeated measurements in a relatively parsimonious way and allows time-varying covariates to be included in the model [61].

Fourthly, even if information about the use psychotropic medications were available, we did not have information about the dosages or length of the use of the psychotropic medications. We also used a very rough assessment of level of care, i.e. domiciliary care or nursing home care at the second and third assessment time point. We did not have detailed information about the degree of domiciliary care that was provided at any assessment time point, but we expect that individuals whose ADL functions decline a great deal will have received more care compared with those who performed better. Lastly, since I-ADL function is often related to mobility [62], it would be fair to criticize our study for not adjusting our analysis of I-ADL functioning to reflect the degree of difficulty with mobility. Including adjustments for the degree of domiciliary care provided and the degree of difficulty with mobility may have nuanced our results further. As a consequence, we cannot rule out that there are confounders that are not reflected in our analyses.

## Conclusion

In a large-scale longitudinal study among older people who receive domiciliary care that included three assessments over a 36-month period, we found that P-ADL and I-ADL functioning declined over time. Furthermore, the worsening of P-ADL and I-ADL functioning was associated with lower baseline cognitive functioning and with a decline in cognitive functioning during follow-up. The degree of decline in I-ADL functioning explained by cognitive functioning decreased during follow-up. Clinicians should pay attention to the associates of P-ADL and I-ADL functioning identified in this study so they can better tailor care, and thus, help older people maintain their level of functioning as long as possible.

## Abbreviations

ATC: The Anatomic Therapeutic Chemical
CDR: Clinical Dementia Rating
CDT: Clock Drawing Test
I-ADL: Instrumental Activities of Daily Living
ICD-10: International Statistical Classification of Diseases and Related Health Problems 10th Revision
IQ-CODE: Informant Questionnaire on Cognitive Decline in the Elderly
NPI: Neuropsychiatric Inventory
MCI: Mild Cognitive Impairment
MMSE: Mini Mental State Examination
P-ADL: Personal Activities of Daily Living
SAS: Statistical Analysis Software
SPSS: Statistical Software Package from IBM
T1: Baseline
T2: The second assessment
T3: The third assessment
